# In Memoriam—Dr. Mahlon Preston

**Published:** 1895-11

**Authors:** 


### IN MEMORIAM—DR. MAHLON PRESTON.

In the death of Dr. Mahlon Preston, of Norristown, Pennsylvania, Homœopathy has lost one of its staunchest friends and closest followers. True to Homœopathy under all its conditions, his sole idea in life was to follow out its strictest principles, and demonstrate its incontestable truth by its careful, patient, and faithful application to the alleviation and cure of the sick.

Throughout his life he was a student. With strong scientific instincts, his attention at the beginning of his career was specially directed to the study of botany, which later he made subservient to his one great object, the curing of the sick.

Perseverance until the final accomplishment of his object was his distinguishing characteristic. This is well shown in an incident related of him by his brother, when at the age of fourteen years. He had resolved to build a working model of a steam engine, though he had scarcely any tools and no materials. He collected together all manner of odds and ends of brass and iron that he happened to meet with, and then out of these unpromising scraps he proceeded to build his engine. Failure after failure attended his attempts. The most desperate efforts of his boyish strength failed to conquer the stubborn metal, yet he never abandoned his project. Month after month he toiled on with varying progress, but with great expenditure of nervous energy and muscular strength, and often with the exhibition of tears. His parents' advice to give up his design went unheeded and he persisted, until at last success crowned his persevering labor, and when the steam was turned on the wheel revolved and his work was done.

The perseverance here exemplified inspired him later in life to the accomplishment of his great purpose to master the homœopathic therapeutics.

An examination of his library shows the presence there of every book issued in any way bearing upon homœopathic materia medica. A closer inspection of the books themselves discovers them loaded with notes, cross references, and various distinguishing pencil marks, all in his own handwriting, and all designed to make more easy and certain the selection of the simillimum.

*The Daily Herald* of Norristown in its issue of Thursday, October 3d, says of him :

" Dr. Mahlon Preston, a leading homœopathic physician of the county, died at his home on East Penn Street about one o'clock on Wednesday afternoon, October 2d, in the fifty-seventh year of his age. He was a pioneer in his school of medicine in this section of Montgomery County, coming to Norristown thirty-three years ago. His death was due to a complication of diseases, in which heart troubles were a prominent feature. He has been ailing nearly a year, but, until recently, was able to drive out occasionally.

" Dr. Preston was born in East Caln, now Valley Township, Chester County, January 22d, 1839. He was a descendant of the well-known family of Friends of that name, his father being Isaac Coates Preston. His mother is still living at an advanced age, being by a singular coincidence a lineal descendant of another family of Prestons of Philadelphia, of which Samuel Preston, Mayor of Philadelphia in 1712, was a well-known member.

" He studied medicine with Dr. J. Bayard Wood, of West Chester, and graduated at the Homœopathic College, now Hahnemann, Philadelphia, in March, 1861. He located for a short time, successively, at Meadville, Spring Centre, and Rome, New York. Then he came to Chester, Delaware County, Pennsylvania, as the assistant of his uncle, Dr. Coates Preston. Finally in August, 1862, he came to Norristown, taking an office on the present site of the Opera House. In 1867 he married Mary, daughter of Judge David Krause. Their children are three: Frederick, Catharine, and Emily Preston.

" On the death of Judge Krause, in 1871, Dr. Preston purchased and removed to the stone cottage on Penn Street, adjoining the Court-house grounds, where the family have since resided. The original building has been much improved, and a roomy office placed in front.

"Dr. Preston made his way as a physician in the face of deep-seated prejudice against what was then the new school of medicine, building up gradually a lucrative practice, which extended miles beyond the limits of Norristown. Several prominent and successful physicians studied the system under his instruction, most of whom enjoy lucrative incomes from their practice in adjoining counties.

"He was looked up to by the younger physicians of the homœopathic school as one of the oldest and most successful practitioners. In 1881 he attended the World's Homœopathic Congress in London, as a delegate, and took a prominent part in its discussions."

He was celebrated in the counties of Montgomery, Chester, Delaware, and Philadelphia for his strict adherence to the principles of Homœopathy; for his fine abilities as a practitioner of these principles, and for his devotion of himself to the one cause of conquering sickness by the application of the most similar remedy.

He founded The Medical Council, an association of physicians who meet to discuss the cases they are treating, and to secure advice from each other in the further treatment of them.

He had a very large practice, and was widely known for his cures of difficult cases. His devotion to his practice was absolute. He would neither drink nor smoke because he feared such habits would incapacitate him for his work. He constantly took regular exercise in his own gymnasium and long walks, the better to keep up his strength.

He was never a perfectly well man, and so was constantly threatened with a premature end. His last illness began in December, 1894, with shortness of breath and tension in the chest. After a number of remedies had been given with but little result, Calcarea-carbonica was prescribed with such success that he believed himself cured. The disease, a complicated one, returned, however, and for nine months a fearful struggle for life was kept up, in which his attending physicians were his brother and the editor of this journal, aided by frequent consultations with Dr. Carleton Smith, of Philadelphia, Dr. J. W. Thomson, of New York, and Dr. Cleveland, of Philadelphia. Through all his sufferings he showed the utmost fortitude, patience, and gentleness, until death closed his career.

---

